# Relationship Among Panoramic Radiography Findings, Biochemical Markers of Bone Turnover and Hip BMD in the Diagnosis of Postmenopausal Osteoporosis

**Published:** 2011-03-30

**Authors:** M. Johari Khatoonabad, N. Aghamohammadzade, H. Taghilu, F. Esmaeili, H. Jabbari Khamnei

**Affiliations:** 1 Assistant Professor, Department of Oral and Maxillofacial Radiology, Dental Faculty, Tabriz University of Medical Sciences, Tabriz, Iran; 2 Assistant Professor, Department of Endocrinology, Imam Reza Hospital, Tabriz University of Medical Sciences, Tabriz, Iran; 3 Postgraduate Student, Department of Oral and Maxillofacial Radiology, Dental Faculty, Tabriz University of Medical Sciences, Tabriz, Iran; 4 Assistant Professor, Department of Statistics, School of Mathematical Sciences, Tabriz University, Tabriz, Iran

**Keywords:** Osteoporosis, Panoramic Radiography, Radiomorphometric Indices, Bone Turnover Biomarkers

## Abstract

**Background/Objective:**

Recent investigations have shown that panoramic radiography might be a useful tool in the early diagnosis of osteoporosis. In addition, bone turnover biochemical markers might be valuable in predicting osteoporosis and fracture risks in the elderly, especially in post-menopausal women. The aim of the present study was to evaluate the relationship among the radiomorphometric indices of the mandible, biochemical markers of the bone turnover and hip BMD in a group of post-menopausal women.

**Patients and Methods:**

Evaluations of mandibular cortical width (MCW), mandibular cortical index (CI), panoramic index (PMI) and alveolar crest resorption ratio (M/M ratio) were carried out on panoramic radiographs of 140 post-menopausal women with an age range of 44-82 years. Hip BMD was measured by DEXA method. BMD values were divided into three groups of normal (T score>-1.0), osteopenic (T score, -2.5 to -1.0) and osteoporotic (T score<-2.5). Serum alkaline phosphatase and 25(OH) D_3_ were measured.

**Results:**

A decrease in MCW by 1 mm increases the likelihood of osteopenia or osteoporosis up to 40%, having taken into consideration the effect of menopause duration. A 1 mm decrease in MCW increased the likelihood of moderate or severe erosion of the lower cortex of the mandible up to 28% by taking age into consideration. The results did not demonstrate a statistically significant relationship between bone turnover markers and mandibular radiomorphometric indices.

**Conclusion:**

Panoramic radiography gives sufficient information to make an early diagnosis regarding osteoporosis in post-menopausal women. Panoramic radiographs may be valuable in the prevention of osteoporotic fractures in elderly women.

## Introduction

Osteoporosis is a prevalent condition all over the world. It is very common in the elderly, especially in post-menopausal women.[1] It is a skeletal disorder which is associated with a decrease in bone mass density, resulting in bone fractures in the affected individuals.[1][2] Osteoporotic fractures are associated with severe problems, including death, in a significant number of patients.[3] It is a predictable condition; therefore, if early diagnosis is made, complications will decrease along with the resultant costs inflicted on the community and general population.

At present, several techniques are used to diagnose osteoporosis. The most common technique is bone densitometry using DEXA (Dual Energy X-ray Absorptiometry) technique.[1][3][4]Since the oral cavity and jaws undergo regular radiographic examinations, it is likely that evaluation of a number of indices on panoramic radiographs might yield useful information regarding bone mass density. These indices are related to the mandible and include the cortical index (CI), the mandibular cortical width (MCW), the panoramic index (PMI) and the alveolar crest resorption ratio (M/M ratio). Several studies have been carried out in this regard, some of which have reported positive relationship between mandibular radiomorphometric indices and BMD.[5][6] On the other hand, some other studies have not reported any relationship between these indices and BMD.[7][8] Furthermore, some studies have attempted to evaluate the relationship between BMD, mandibular radiomorphometric indices and bone turnover biomarkers leading to contradictory results.[9] Therefore, the aim of the present study was to evaluate the relationship among mandibular radiomorphometric indices, BMD and bone biomarkers in post-menopausal women.

## Patients and Methods

The subjects were selected from post-menopausal women who had been referred from the Endocrinology and Rheumatology Clinic for BMD evaluation and densitometry by DEXA technique to the Densitometry Department at Sina Hospital, Tabriz. The subjects were selected from those patients who were ready to undergo a panoramic radiographic examination for the evaluation of their dental status. The subjects were 44-82 year olds who had not experienced menstruation for at least the last year (range, 1-32 years) before the densitometry procedure. Subjects with a history of corticosteroid and anticonvulsant consumption, those suffering from endocrine disorders or blood malignancies, those suffering from chronic renal disease or with a history of hormone therapy (estrogen or pharmaceutical supplements) and finally those with a history of hysterectomy were excluded from the study.[1] On the whole, 140 subjects who volunteered to take part in the study were evaluated. All the subjects signed informed written consent forms. The study was evaluated and approved by the Research and Ethics Committee of Tabriz University of Medical Sciences.

Hip BMD was measured by DEXA technique using Hologic QDR 4500/Acclaim equipment. BMD values were classified into three groups of normal (T score>-1), osteopenic (T score from -2.5 to -1) and osteoporotic (T score<-2.5) according to WHO criteria. Subjects’ age and the duration of the post-menopausal period were recorded at the densitometry session.

Simultaneous with the densitometry procedure, the subjects were referred to the laboratory for alkaline phosphatase, calcium, phosphorus and 25(OH) D_3_ evaluations. Alkaline phosphatase (ALP) and 25(OH) D_3_ were measured by enzyme immunoassay and elect rochemoluminescence, respectively, by Elecsys (2010) equipment.

Subsequent to the densitometry procedure, all the subjects underwent a panoramic radiographic examination while all the protective procedures against radiation, including the use of lead aprons were observed. Planmeca Promax (Helsinki, Finland) radiography equipment was used for the purpose with a total filtration of 2.5 mm of aluminum at 64-74 kVp and 160-265 mAs depending on the age and size of the subjects. An automatic processing machine was used to develop films at standard conditions. Film speed was 400. All the standard criteria for head positioning, film density and patient positioning were observed to achieve the best film quality. The number of teeth was assessed; third molars were not taken into account. Radiomorphometric indices were measured on both sides of the mandible by two oral radiologists who were blind about the results of the bone densitometry procedure.

Morphologic classification of the lower mandibular cortex was carried out by observing it distal to the mental foramen bilaterally; the lower cortex of the mandible was classified into C1, C2, and C3 groups according to the method used by Klemetti et al.:[2][10][11]

C1: The endosteal margin was smooth and clear on both sides of the cortex.

C2: The endosteal margin had semi-lunar defects.

**Fig. 1:**
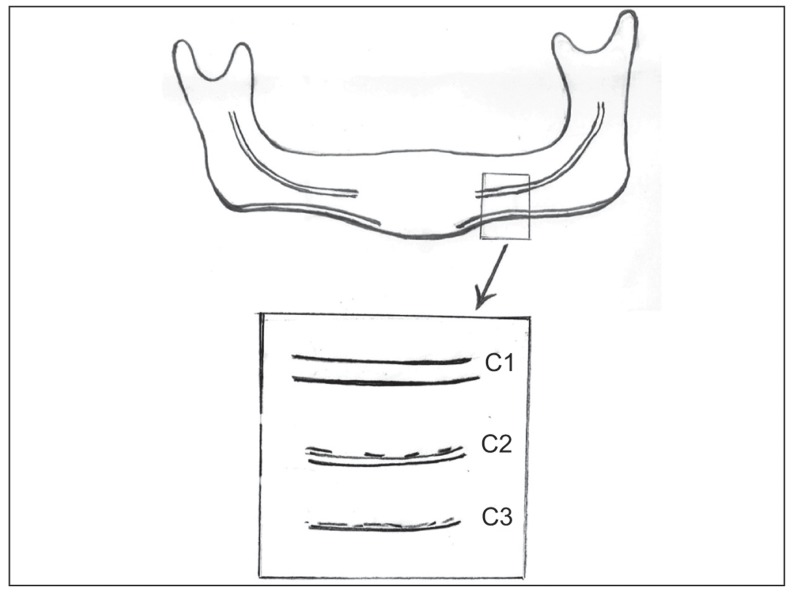
Schematic view of the panoramic radiography. Classification of the morphology of the lower cortex of the mandible (CI: C1, C2, C3)

C3: The endosteal cortex had clearly visible porosity (Fig. 1).

**Fig. 2:**
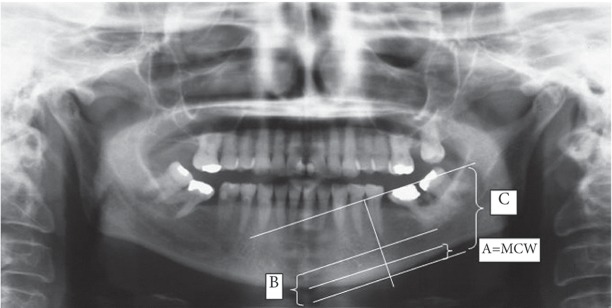
Panoramic radiography. Measurements of the quantitative indices. For more details refer to the text (MCW=A, PMI= A/B, M/M ratio=C/B).

MCW was bilaterally measured at the mental foramen region by Dentaraum orthodontic calipers and the means were recorded. A line was drawn parallel to the longitudinal axis of the mandible tangential to the lower border of the mandible; then another line was drawn parallel to the first one from the inner surface of endosteum. The distance between these two lines at the mental foramen region was considered MCW.[2][9][12][13] PMI was calculated by dividing the MCW by the distance between the center of the mental foramen and the lower cortex of the mandible (Fig. 2).

M/M ratio was calculated by dividing the overall height of the mandible by the distance between the center of the mental foramen to the lower border of the mandible. MCW, PMI and M/M ratio were measured bilaterally in milimeters and their means were recorded.[2][7]

The SPSS for windows 16 software was used for statistical analysis. Data regarding bone densitometry and biochemical markers and radiographic measurements were analyzed by descriptive (frequency, percentage, means, standard deviations) statistics. For the evaluation of the relation between qualitative indices and BMD, the chi-square test was used. For quantitative indices one way analysis of variance (ANOVA) was used. A paired 2 tailed test was used to evaluate inter-examiner agreement in measurements. Linear regression analysis was used to predict the width of mandibular cortex based on age and duration of the post-menopausal period. Logistic regression was used to evaluate the effect of mandibular cortex thickness on BMD and cortical erosion.

## Results

The mean age of the subjects was 59.76 years (44-82 years). The median duration of the post-menopausal period was 10.5 years. The characteristics of the subjects are presented in Table 1. No statistically significant differences were observed in MCW, PMI and M/M ratio measurements between the two observers (all p values>0.05). The kappa value for interobserver agreement was 0.80 for the mandibular cortical index (SE= 0.048).

**Table 1:** Comparision of Characteristics Among Patients Classified Based on Hip BMD

	**Hip BMD^a^**			**P Value**
	**Normal**	**Osteopenic**	**Osteoporotic**	
Age (years)	57.08 (6.26)	60.64 (7.04)	67.54 (9.13)	0.001
Menopause Duration (years)	9.73 (6.02)	12.06 (7.51)	17.18 (8.12)	0.004
MCW	3.81 (0.64)	3.43 (0.76)	2.84 (0.58)	0.001
PMI	0.26 (0.06)	0.25 (0.06)	0.22 (0.07)	0.114
M/M Ratio	1.93 (0.47)	1.75 (0.45)	1.67 (0.47)	0.048
Number of Lost Teeth	18.21 (9.87)	21.79 (9.41)	21 (10.08)	0.1

^a^ Mean (SD)

Two-by-two comparisons of the means of the age between the groups revealed statistically significant differences. The difference in the means of the duration of post-menopausal period was statistically significant only between the normal and osteoporotic groups (Table 1).

### Hip Densitometry Results 

Fifty-six subjects (40%) had normal bone density; 73 subjects (52.1%) were osteopenic; and 11 subjects (7.9%) were osteoporotic.

### Radiomorphometric Indices and BMD

MCW showed statistically significant differences between the three groups (p<0.05). A post hoc Tukey test revealed two-by-two differences between the groups. One-way ANOVA showed statistically significant differences in M/M ratios between the groups; however, a post hoc Tukey test did not emonstrate two-by-two differences between the groups. No significant difference was found in PMI between the groups. There were no statistically significant differences in the number of the teeth lost between the groups (p>0.05) (Table 1).

Chi-square test demonstrated statistically significant difference in the quality of mandibular cortex (cortical index: CI) between the groups (p=0.012) (Table 2). Then C2 and C3 were grouped together. Table 3 demonstrates significant differences between two groups.

**Table 2:** Cortical Index of the Study Population Regarding Hip BMD

**Hip BMD [No. (%)]**				
	**Normal**	**Osteopenic**	**Osteoporotic**	**Total**
C1	32 (57%)	25 (34.24%)	3 (27.27%)	60 (42.85%)
C2	24 (43%)	43 (58.9%)	6 (54.54%)	73 (52.15%)
C3	0 (0%)	5 (6.86%)	2 (18.18%)	7 (5%)
Total	56 (100%)	73 (100%)	11 (100%)	140 (100%)

**Table 3:** Comparison of Characteristics Between Two Groups (C1 vs. C2 and C3)

	**C1^a^**	**C2 and C3^a^**	**P Value**
Age (year)	56.58 (5.72)	62.14 (7.79)	<.0001
Menopause Duration (years)	8.85 (6.34)	13.53 (7.24)	<.0001
MCW	4.04 (0.58)	3.18 (0.63)	<.0001
Ca (mg/dl)	9.37 (0.55)	9.34 (0.56)	0.74
Ph (mg/dl)	3.82 (0.47)	3.79 (0.41)	0.62
ALP (IU/L)	1.87 (53.54)	1.85 (66.1)	0.80
25 (OH) D3 (ng/m1)	24.29 (16.4)	24.55 (19.77)	0.93

^a^ Mean (SD)

### Bone Turnover Biomarkers

Means of alkaline phosphatase, calcium, phosphorus and vitamin D_3_ levels are presented in Table 4. There were no statistically significant differences in the means of biomarkers between the three groups.

**Table 4:** Comparison of Mean Biochemical Marker Levels Among Study Population Regarding Hip BMD

	**Normal^a^**	**Osteopenic^a^**	**Osteoporotic^a^**	**P Value**
ALP (IU/L)	189.86±62.75 (68-385)	183.67±60.75 (89-355)	191.45±62.84 (102-312)	0.824
Calcium (mg/dl)	9.3±0.56 (7.7-10.6)	9.42±0.56 (8.2-10.8)	9.20±0.41 (8.5-9.8)	0.320
Phosphorous (mg/dl)	3.83±0.43 (2.62-5.1)	3.78±0.44 (2.7-4.8)	3.74±0.42 (3-4.5)	0.695
25 (OH) D3 (ng/m1)	20.80±15.00 (4-65.7)	25.45±16.92 (4-66.8)	22.39±14.71 (4-53)	0.268

^a^ [Mean±SD] (range)

### Regression Analysis

By carrying out a linear regression analysis between the MCW, age and duration of post-menopausal period the following were obtained:

MCW= 5.72 – 0.037 (Age)

MCW= 3.95 – 0.035 (post-menopausal period duration)

It means there is a 0.035-mm decrease in MCW with a 1-year increase in the post-menopausal period duration. Table 5 presents the results of logistic regression model. Osteopenic and osteoporotic subjects were classified in one group in order to carry out this test. Of all the factors involved only MCW was statistically significant, i.e. a 1-mm decrease in MCW increases the likelihood of osteopenia or osteoporosis up to 40%.

**Table 5:** The Association Between Different Characteristics of Mandibular Bone and Hip BMD Adjusted for Years of Elapsed Menopause

	**T- Score at Hip Bone**		
	**OR^a^**	**95%CI^b^**	**P Value**
MCW Decrease per 1mm	1.38	1.1-1.99	0.008
M/M ratio Increase per 1mm	0.61	0.27-1.37	0.235
PMI Increase per 0.1mm	0.8	0.009-1.2	0.453

^a^ Odds Ratio

^b^ Confidence Interval

In the evaluation of cortical index (CI), C2 and C3 were grouped together. In this case too, only MCW was statistically significant. A 1-mm decrease in MCW increased the likelihood of moderate to severe erosion up to 28% (Table 6). No relationship was noted between biomarkers and radiomorphometric indices or BMD.

**Table 6:** The Association Between Different Characteristics of Mandibular Bone and Hip BMD Adjusted for Age

	**T- Score at Hip Bone**		
	**OR^a^**	**95%CI^b^**	**P Value**
MCW Decrease per 1mm	0.11	0.04-0.32	<0.001
M/M ratio Increase per 1mm	1.14	0.3-4.38	0.84
PMI Increase per 0.1mm	0.001	0.00-10.65	0.14
Number of Teeth Loss Increase per 1	1.03	0.97-1.1	0.307

^a^ Odds Ratio

^b^ Confidence Interval

## Discussion

Panoramic radiography is an imaging technique which is used by dental practitioners and some medical specialists. The oral cavity undergoes radiographic examinations more than other parts of the body. Radiomorphometric indices of the mandible on panoramic radiographs can be used to gain information about bone density.[2][14]

In the present study, mandibular cortical width in the mental foramen area were different in the three normal, osteopenic and osteoporotic groups, which is consistent with the results of some previous studies, including studies carried out by Taguchi et al.[9] and Vlasiadis et al.,[2] though both studies were based on lumbar densitometry. In addition, Mahl et al. demonstrated significant difference in the value of mandibular cortical width among these three groups.[13] In the present study a 1-mm decrease in MCW resulted in a 40% increase in the likelihood of osteopenia or osteoporosis, when the duration of post-menopausal period was taken into account. In the study carried out by Vlasiadis et al., a 1-mm decrease in MCW increased the odd of osteopenia or osteoporosis up to 43%.[2] In addition, in another study carried out by the Vlasiadis in 2008, a 1-mm decrease in MCW increased the odds of osteopenia or osteoporosis up to 47% in postmenopausal women.[15] Some studies have not reported any relationship between hip BMD and MCW.[16] In our study, no statistically significant differences were noted in PMI and M/M ratios between the groups (p>0.05), which is consistent with the results of a study carried out by Vlasiadis et al.[2]

CI was significantly different in the three groups. Moderate to severe cortical erosion (C2 and C3) significantly increased the likelihood of osteopenia or osteoporosis. Furthermore, with a 1-mm decrease in MCW, the likelihood of moderate to severe cortical erosion increased up to 28%, when age was taken into account. In addition, the means of age, duration of the post-menopausal period and MCW were significantly different between the two (C1) and (C2 and C3) groups. When the cortex was classified as C2 or C3, the age and the post-menopausal period duration increased and MCW decreased. There was a relationship between the quality of the cortex and the quantitative index of the mandible. When the quality of the cortex is low, the value of the MCW decreases. Zlataric et al. demonstrated that the patients with severe erosion of the cortex had significantly lower BMD values in comparison to other patients.[17] In a study carried out by Yasar et al., CI was considered an important criterion to distinguish osteoporotic and non-osteoporotic subjects from each other.[7] However, Delvin et al. have reported that the accuracy of CI is less than that of MCW.[18]

There were no significant differences in the number of teeth lost between the three groups. The effect of tooth loss on the lower cortex of the mandible has not been elucidated yet. Also, Taguchi et al.,[9] did not find any correlation between the loss of teeth and MCW as age increased. Alkaline phosphatase, calcium, phosphorus and 25(OH) D_3_ levels were not significantly different between the three groups. Furthermore, no relationship was observed between 25(OH) D_3_ and other biomarkers on one hand and cortical erosion on the other. However, in a study carried out by Vlasiadis, alkaline phosphate levels were different in different groups; they reported that a 1-unit increase in the alkaline phosphatase level increases the odds of osteopenia or osteoporosis up to 14%.[15]

Furthermore, in a study carried out by Taguchi, bone biomarkers such as NTX had a significant relationship with the morphology of mandibular cortex.[19] Some other studies have not reported a relationship between biomarkers and BMD.[20] 25(OH) D_3_ levels were less than normal in the three groups, with no statistically significant differences. Since vitamin D deficiency might result in serious complications, such as bone fracture, immunologic disorders and an increased risk of infection, methods such as supplementation of food material with vitamin D, sufficient exposure to sunlight and use of pharmaceutical supplements can be useful in preventing such problems.

In conclusion, panoramic radiography is an imaging technique commonly used by dentists which gives a lot of information. The present study evaluated the radiomorphometric indices of the mandible. A significant relationship was noted between CI and MCW on one hand and hip bone density on the other. Furthermore, a relationship was noted between the age and duration of the post-menopausal period on one hand and MCW on the other. We could not evaluate the relation of other biomarkers such as NTX and BMD. However, further studies about the relationship among bone turnover biomarkers and panoramic radiography findings should be performed. Practitioners may play a great role in the screening and early diagnosis of osteoporosis by examining panoramic radiographs and its radiomorphometric indices.
